# A High Burden of Respiratory Syncytial Virus Associated Pneumonia in Children Less than Two Years of Age in a South East Asian Refugee Population

**DOI:** 10.1371/journal.pone.0050100

**Published:** 2012-11-20

**Authors:** Claudia Turner, Paul Turner, Verena Cararra, Naw Eh Lwe, Wanitda Watthanaworawit, Nicholas P. Day, Nicholas J. White, David Goldblatt, François Nosten

**Affiliations:** 1 Shoklo Malaria Research Unit, Mae Sot, Thailand; 2 Mahidol-Oxford Tropical Medicine Research Unit, Bangkok, Thailand; 3 Centre for Tropical Medicine, University of Oxford, Oxford, United Kingdom; 4 Immunobiology Unit, Institute of Child Health, University College London, London, United Kingdom; Instituto de Higiene e Medicina Tropical, Portugal

## Abstract

**Background:**

Pneumonia is a major cause of childhood mortality and morbidity approximately 1.6 million deaths and 150 million episodes occur annually in children <5 years. Respiratory syncytial virus (RSV) may be responsible for up to 25% of cases and 12% of deaths making it an important potential vaccine target, although data from South East Asia is scarce.

**Methods:**

We followed a birth cohort of Burmese refugee children, born over a one year period, for two years. Pneumonia episodes were diagnosed using WHO criteria. A chest radiograph, nasopharyngeal aspirate and non-specific markers of infection were taken during each episode.

**Results:**

The incidence of RSV-associated pneumonia was 0.24 (95% CI 0.22–0.26) episodes per child year. All children with pneumonia received antibiotic treatment, following WHO guidelines. The highest incidence was in the 2–12 month age group. The commonest diagnosis in a child with RSV-associated pneumonia was non-severe pneumonia (239/362∶66.0%), however the incidence of RSV-associated severe or very severe pneumonia was 0.08 (95% CI 0.01–0.10) episodes per child year. Birth in the wet season increased the risk of severe disease in children who had their first episode of RSV-associated pneumonia aged 2–11 months (OR 28.7, 95% CI 6.6–125.0, p<0.001). RSV episodes were highly seasonal being responsible for 80.0% of all the pneumonia episodes occurring each October and November over the study period.

**Conclusions:**

There was a high incidence of RSV associated pneumonia in this refugee population. Interventions to prevent RSV infection have the potential to reduce the incidence of clinically diagnosed pneumonia and hence unnecessary antibiotic usage in this population.

## Introduction

Of the 150 million episodes of childhood pneumonia that occur each year, it is estimated that approximately a quarter are caused by respiratory syncytial virus (RSV) [1], [2]. This virus is also responsible for up to 22% of childhood pneumonia hospitalizations and 12% of pneumonia deaths [2], [3]. These proportions may increase with the widespread introduction of the *Haemophilus influenzae* type B and the *Streptococcus pneumoniae* conjugate vaccines [4].

In 1992, WHO and UNICEF launched the Integrated Management of Childhood Illness (IMCI) strategy. This aimed to provide a framework for the management of major childhood illnesses in resource poor community settings [5]. Pneumonia is diagnosed and the severity assessed in a child with cough or difficulty breathing based on two signs: chest indrawing and fast respiratory rate [6]. In determining the thresholds for respiratory rate, a high sensitivity was chosen at the expense of specificity. [7] For individuals, this approach is beneficial; a child with bacterial pneumonia is more likely to be treated with antibiotics. However, there is a cost to the community: that of antibiotic resistance secondary to the inappropriate use of antibiotics [8]. Additional clinical signs with the potential to increase specificity should be investigated.

A case control study performed in Alaska identified viral pathogens in 90% of children hospitalised for pneumonia and in 52% of non-pneumonia controls. However, RSV was significantly more common in hospitalized cases and was the most commonly isolated virus associated with pneumonia [9]. A more recent case control study performed in Kenya showed that RSV isolation in the nasopharynx was associated with severe pneumonia (OR 12.5, 95% CI 3.1–51.5) [10]. These studies demonstrate that many cases of WHO defined pneumonia are associated with RSV infection and these may represent a cohort of children who receive antibiotics inappropriately.

Respiratory syncytial virus is an enveloped RNA virus belonging to the Paramyxovirus family, with two antigenic subgroups A and B. It can cause disease ranging from bronchiolitis to very severe pneumonia [11]–[13]. RSV infection is highly seasonal with outbreaks in the winter or spring in temperate climates, in more tropical areas outbreaks are common in the wet season and maybe more prolonged [11], [12], [14].

An RSV vaccine has the potential to decrease both morbidity and mortality from childhood pneumonia. Unfortunately, development of a vaccine to protect against RSV has proven problematic [15].

The aims of this study were to determine the incidence and seasonality of WHO defined clinical pneumonia associated with RSV in a Burmese refugee population and to establish whether any clinical signs were predictive of RSV infection.

## Methods

### Study Site

The study was conducted in Maela, a camp for displaced persons located 5 km east of the Myanmar (Burma) border in Northwest Thailand. Maela is a densely populated camp with approximately 45,000 people living in 10,000 houses in an area of 4 km^2^. The population in the camp is young, with half being under 18years of age.

Health care in the camp is provided predominately by the non-governmental organisation Premiere Urgence-Aide Médicale Internationale (PU-AMI), though the Shoklo Malaria Research Unit (SMRU) has been providing medical and obstetric care for this population since 1986 and was responsible for providing the medical care for all study participants.

The SMRU clinic in Maela is run by local refugee staff under the supervision of two expatriate doctors. The majority of the staff have no formal medical training but are trained “on the ground” by the expatriate doctors from SMRU. In preparation for this study, locally appropriate guidelines for the diagnosis and treatment of common childhood illnesses were developed based on the IMCI. These guidelines and examination skills were taught to the medical staff. Examination skills focussed on the recognition of the sick infant and child, taking vital signs (in particular respiratory rate) and chest auscultation. Throughout the study period inter-observer variability in respiratory rate assessment was checked. Briefly, three supervisors were nominated and, individually, the respiratory rates of ten children were counted at the same time as the study paediatrician and were compared. A mean difference of less than one breath per minute (BPM) was observed. Subsequently, all staff who were responsible for taking the vital signs of the study children were assessed at several time points throughout the study to ensure accuracy.

### Climate

The climate at Maela is tropical, with temperatures ranging from 15°C–45°C and the monsoon occurring from May to October. Seasons were defined as hot (March – May), wet (June – October) and cool (November – February). Meteorological data was obtained from the Thai government weather reporting station at Mae Sot, a town close to Maela.

### Study Design

We followed a cohort of 965 infants from birth until two years of life. Starting in 2007, women following antenatal care at the SMRU clinic were enrolled at 28–30 weeks gestation over a one year period. They were asked to return to the clinic with their infant for monthly follow up visits, where assessment of health status, growth and development, and review of illnesses and antibiotic use since the previous visit were performed. Mothers were also asked to return with their child any time the child was unwell.

### Pneumonia Episodes

WHO criteria for the diagnosis of clinical pneumonia were used: pneumonia, severe pneumonia and very severe pneumonia were defined by clinical signs [16]. Briefly, children with cough or difficulty breathing and a fast respiratory rate (<2months ≥60 breaths per minute; 2 months –1 year ≥50 breaths per minute and >1 year ≥40 breaths per minute), but no severe signs, were diagnosed as having pneumonia. Those children with cough or difficulty breathing and chest indrawing were diagnosed as having severe pneumonia, and those fulfilling the severe pneumonia criteria but also with cyanosis or inability to suck were diagnosed with very severe pneumonia. Any infant or child who fulfilled the criteria for pneumonia of any severity, but who had wheeze on chest auscultation, was given a trial of inhaled bronchodilator prior to antibiotic treatment. A new episode of pneumonia was defined as occurring if there were 14 symptom free days following the last pneumonia episode [17]. All children received treatment following the recommendations in the WHO pocket book of hospital care for children [18]. All children with a clinically diagnosed pneumonia and who had RSV detected in their NPA were diagnosed as having RSV associated pneumonia.

Children diagnosed with pneumonia had pulse oximetry performed (Hand –held pulse oximeter, model 512, Respironics), a complete blood count (CBC) (PocH-one 100i, Sysmex), C-reactive protein (CRP) (NycoCard, Axis-Shield), nasopharyngeal aspirate (NPA), and a chest radiograph (CXR) taken.

Based on work by Mahdi et al, a high CRP was defined as ≥40 mg/L [19]. A high neutrophil count was defined as being greater than the upper limit of normal (>7.5×10^9^/L) [20].

NPAs were tested for influenza A, influenza B, respiratory syncytial virus (RSV), human metapneumovirus (hMPV) and adenovirus by rRT-PCR as described elsewhere [21]. Briefly, after collection NPAs (in 1 mL viral transport medium, VTM) were stored in a refrigerator on site (supplied with a backup electrical generator) and transported, in a cool box, daily to the laboratory where they were stored at −80°C prior to testing. Viral nucleic acid was extracted from 140 µL thawed VTM using the QIAamp RNA virus kit (Qiagen), following the manufacturers protocol. RSV RNA was detected by singleplex real-time RT-PCR: a specimen was considered positive if the cT value was <40 cycles in the presence of appropriate run control results. Low positive specimens (defined as cT value of 35–39) were repeated to confirm the initial result. A human RNAseP PCR assay was used to determine the adequacy of extraction and absence of PCR inhibitors in all NPA specimens.

Chest radiographs were interpreted by two clinicians using the WHO standardised method and were given the diagnosis of primary endpoint pneumonia (PEP), other infiltrate (OI), normal or uninterpretable [22]. These readings were compared and x-rays with conflicting results were read by a third clinician and the majority diagnosis used. Where there was no agreement between any of the three readers, the CXR was deemed uninterpretable.

### Data Management and Statistical Methods

Data were double entered into Access 2003 databases (Microsoft) and systematically checked for errors. All analyses were performed using Stata/IC 12.1 (StataCorp). Continuous variables were described by the median and inter-quartile range (IQR); comparisons between groups were made using the Wilcoxon rank sum test. Incidence rates were analysed using Poisson regression and groups compared by incidence rate ratios (IRR). For univariate analyses two by two tables were constructed and association tested by the chi-squared test. Odds ratios were calculated using logistic regression. Those factors with a significant p-value (<0.05) were included in a multivariate logistic regression model.

### Ethics Statement

Ethical approval was granted by the Ethics Committee of The Faculty of Tropical Medicine, Mahidol University, Thailand (MUTM 2009-306) and the Oxford Tropical Research Ethics Committee, Oxford University, UK (031-06). All women gave written informed consent to participate in the study.

## Results

### Population

Of the 965 infants included in the cohort, 955 were live born; the neonatal mortality rate was 26.2 per 1000 live births and the infant mortality rate 30.4 per 1000 live births. Only one child died of complications of pneumonia (in addition to a congenital abnormality). Relocation of refugees to the USA led to 290 infants being lost to follow up over the two year study period. However, there were a total of 18,449 follow up visits, with a mean of 19 follow up visits per child.

### Incidence

There were a total of 1,085 episodes of clinical pneumonia diagnosed in the cohort and RSV was detected in 362 (33.4%) of these episodes. The median RSV cT value in those NPA specimens considered positive was 18.1 (IQR 15.5–23.4) and only 6.4% (23/362) had cT values ≥35. The incidence of RSV-associated pneumonia was 0.24 (95% CI 0.22–0.26) episodes per child year. The highest incidence was 0.26 (95% CI 0.23–0.30) episodes per child year in the 2–11 month age group, compared with 0.23 (95% CI 0.20–0.27) in the ≥12 months age group (95% CI 0.20–0.27) and 0.17 (95% CI 0.11–0.24) in the under two month age group. However, RSV was detected in 26/48 (54.2%) of all the pneumonia episodes that occurred in children less than two months of age.

In the younger age group there were more boys than girls (57.7 vs 42.3%) but this did not reach statistical significance (p = 0.4). There was also no statistical association between the age at which the child or infant had RSV-associated pneumonia and birth weight or gestation, although there was a trend to significance between developing pneumonia aged 2–11 months and being born prematurely (p = 0.06) (Table 1).

**Table 1:** Description of infants with a first episode of RSV associated pneumonia, by age group.

	<2 months (N = 26)	2–11 months (N = 171)	≥12 months (N = 101)	
**Sex**				
Male (%)	15 (57.7)	90 (52.6)	42 (41.6)	
Female (%)	11 (42.3)	81 (47.4)	59 (58.4)	
**Birth weight (kg)**				
Median (IQR)	3.085	2.865	2.910	
	(2.750–3.160)	(2.580–3.195)	(2.650–3.130)	
Low birth weight (%)	3/26 (11.5)	31/168 (18.5)	15/101 (14.9)	
**Gestation (weeks)**				
Median (IQR)	39.1	39.1	39.4	
	(38.2–40.3)	(38.1–39.6)	(38.3–40.0)	
Premature (%)	1/26 (3.9)	16/169 (9.5)	4/101 (4.0)	
**Season of birth**				Total
Hot (%)	0 (0.0)	51 (29.8)	19 (18.1)	70 (23.5)
Wet (%)	26 (100.0)	46 (26.9)	57 (56.4)	129 (43.3)
Cool (%)	0 (0.0)	57 (56.4)	25 (24.8)	99 (33.2)
**Clinical disease**				
Pneumonia (%)	2 (7.7)	113 (66.1)	79 (78.2)	194 (65.1)
Severe pneumonia (%)	12 (46.2)	42 (24.6)	13 (12.9)	67 (22.5)
Very severe pneumonia (%)	12 (46.2)	16 (9.4)	9 (8.9)	37 (12.4)
**CXR**				
Normal (%)	5 (25.0)	12 (7.9)	1 (1.0)	18 (6.7)
Other infiltrate (%)	14 (70.0)	97 (63.8)	61 (62.2)	172 (63.7)
Primary endpoint pneumonia (%)	1 (5.0)	43 (28.3)	36 (36.7)	80 (29.6)

### Seasonality

**Figure 1:**
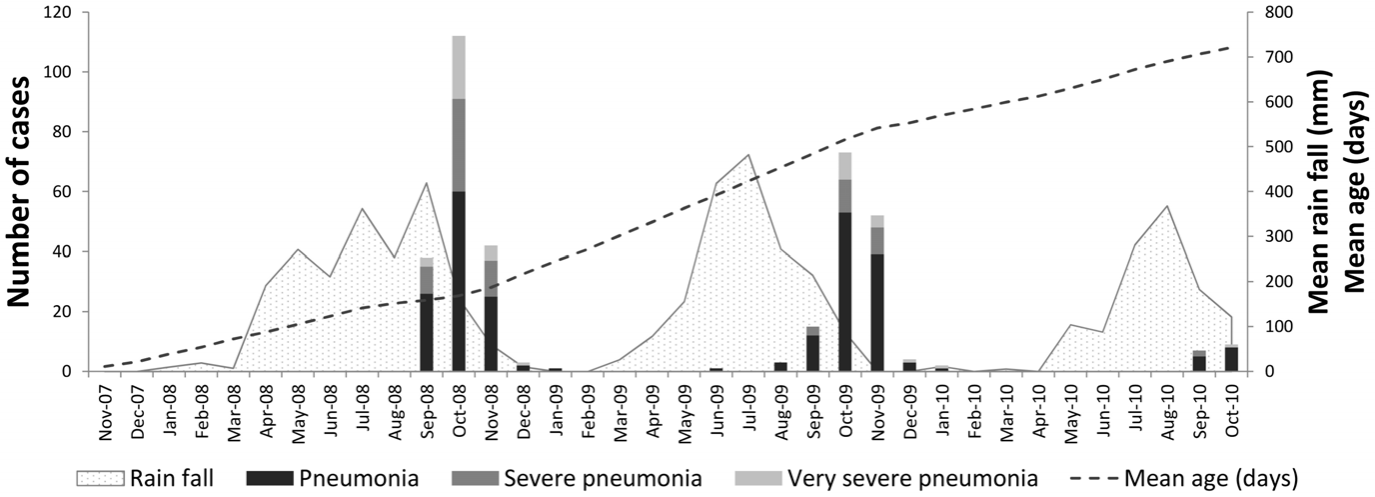
RSV associated pneumonia cases by season and severity. The mean age of the cohort (dashed line) and the mean monthly rainfall (grey shaded area) are highlighted.

RSV episodes were highly seasonal, occurring predominantly in October each year, just after the peak rainfall (Figure 1). Of the pneumonia episodes that occurred each October and November over the study period, 80.0% (295/369) were associated with RSV. All infants who had an episode of RSV-associated pneumonia before two months of age were born in the wet season. Being born in the wet season also increased the risk of severe disease in children who had their first episode of RSV associated pneumonia aged 2–11 months (OR 28.7, 95% CI 6.6–125.0, p<0.001), but not in those children aged ≥12 months.

### Clinical Disease

The most common diagnosis in a child with RSV-associated pneumonia was non-severe pneumonia (239/362∶66.0%), followed by severe pneumonia (77/362∶21.3%) and very severe pneumonia (46/362∶12.7%).

RSV-associated pneumonia was more severe in the youngest age group, with 24/26 (92.3%) of infants less than two months having severe disease. There was no association with severity of the pneumonia and the 2–11 month age group (p = 0.7). In the ≥12 month age group there was an association with non-severe disease, with 116/155 (74.8%) episodes being non severe pneumonia (p = 0.002).

### Multiple Episodes

**Figure 2:**
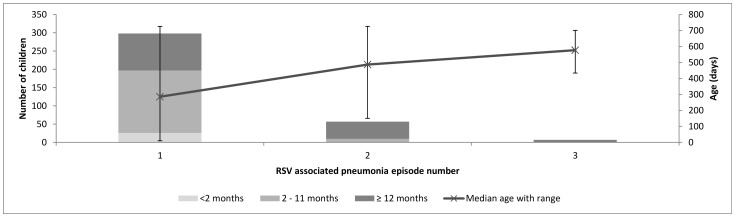
RSV associated pneumonia episodes by age group. In the above graph the bars show the number of children who had 1, 2 and 3 episodes of RSV associated pneumonia by the age group in which they occurred. The median age at which the episode number occurred is indicated by the line with 95% CI demonstrated.

Multiple episodes of RSV-associated pneumonia were common. Two hundred and ninety eight children had at least one episode: 50 of these (16.8%) had two episodes and 7/298 (2.3%) had three episodes. Subsequent episodes of RSV-associated pneumonia occurred in the older age groups, with no second episode occurring in infants less than two months of age and no third episode occurring in children aged less than one year (Figure 2). The first episode of RSV associated pneumonia was not associated with more severe disease as compared to subsequent episodes (p = 0.4).

Multiple episodes of clinical pneumonia were also common: 206/298 (69.1%) of RSV-associated pneumonia represented the child’s first episode of pneumonia, although the range was the first to the seventh episode.

### Clinical Features

Clinical signs associated with lower respiratory tract infection were analysed to determine whether they were predictive of RSV infection. Of the 12 clinical signs assessed, five were significantly associated with RSV detection (p<0.05). In a multivariate logistic regression model, controlling for age, three remained significant: fever and tachycardia on admission were positively associated with RSV infection, as was the presence of bilateral chest signs (crepitations and/or wheeze) on auscultation (Table 2). Bilateral chest signs had a sensitivity of 75.0% (95% CI 70.1–79.5) for a clinically diagnosed pneumonia being associated with RSV but a specificity of only 36.8% (95% CI 33.2–40.5). Tachycardia on admission had the highest specificity for pneumonia to be associated with RSV: 88.4% (96% CI 85.8–90.6) but a sensitivity of only 18.1% (95% CI 14.3–22.5) (Table 3).

**Table 2:** Analysis of clinical signs for associations with RSV infection.

	Number of RSV positive	% RSV +ve with sign	Univariate analysis	Multivariate analysis		
			p-value	Odds Ratio	95% CI	p-value
Fever on admission	120/362	33.2	0.009	1.4	1.0–2.0	0.04
Tachycardia	65/359	18.1	0.004	1.6	1.0–2.4	0.04
Hypoxia	19/310	6.1	0.02	2.1	0.9–4.7	0.06
Prolonged CRT	9/334	2.7	0.1	0.8	0.2–3.2	0.8
Grunting	10/362	2.8	0.08	1.1	0.3–1.6	0.9
Tracheal tug	38/362	10.5	0.2	0.8	0.5–1.4	0.4
Nasal flaring	38/362	10.5	0.3	0.7	0.5–1.4	0.4
Head Bobbing	19/362	5.3	0.09	0.8	0.4–1.9	0.7
Chest in-drawing	153/350	43.7	<0.001	1.3	0.7–2.5	0.4
Unilateral crepitations	44/356	12.4	0.4	1.4	0.9–2.3	0.2
Unilateral wheeze	19/348	5.5	0.8	1.4	1.0–2.1	0.03
Bilateral crepitations or wheeze	261/697	37.5	<0.001	2.0	1.3–2.9	0.001

**Table 3:** The sensitivity, specificity, positive predictive value and negative predictive value of clinical features significantly associated with RSV associated pneumonia.

	Sensitivity	Specificity	Positive predictivevalue %	Negative predictivevalue
	% (95% CI)	% (95% CI)	(95% CI)	% (95% CI)
Fever on admission	33.1 (28.3–38.3)	74.4 (71.0–77.6)	40.0 (34.4–45.8)	68.4 (65.0–71.7)
Tachycardia	18.1 (14.3–22.5)	88.4 (85.8–90.6)	44.2 (36.0–52.6)	67.9 (64.8–71.0)
Bilateral crepitations orwheeze	75.0 (70.1–79.5)	36.8 (33.2–40.5)	37.4 (33.8–41.2)	74.5 (69.5–79.0)

In all age groups, the commonest radiological finding was OI, 172/270 (63.7%). In 102 (30.1%) children, PEP was seen on their chest radiograph, and this was a more common radiological finding with increasing age (p = 0.008) (Table 1).

In a multivariate logistic regression model controlling for age, a combination of high CRP and neutrophil count was not associated with RSV infection (OR 0.81, 95% CI 0.51–1.29, p = 0.4). However in those children with RSV infection, there was a significance association between PEP on chest radiograph and a high CRP and neutrophil count (OR 2.3, 95% CI 1.5–3.5, p<0.001), suggesting that these cases had a secondary bacterial superinfection.

## Discussion

We followed a birth cohort of refugee children on the Thailand-Myanmar border for the first two years of life, in a study encompassing three RSV seasons. The WHO criteria for the diagnosis of pneumonia were applied rigorously to all children presenting unwell to the clinic with a cough or difficulty breathing. RSV was detected in approximately one third of all clinical pneumonia cases giving an incidence of 0.24 episodes per child year. The incidence of RSV detection associated with severe pneumonia or very severe pneumonia was 0.08 episodes per child year. These figures are considerably higher than those reported by Nokes and colleagues from their study conducted in Kenya, a study with comparable characteristics to that reported here. They reported an incidence of RSV-associated lower respiratory tract infection (LRTI) of 0.09 per child year and severe LRTI as 0.04 episodes per child year. However the authors used direct fluorescent antibody detection methods for RSV and not PCR which may explain the higher incidence reported in our study [12]. There are no published studies reporting community RSV associated pneumonia incidence from Thailand or Myanmar. This difference in incidence could be explained by the specific characteristics of our study population. Maela camp is crowded, with the majority of nuclear families living in small one room houses and all sleeping in the same bed. Crowding has been shown to be a risk factor for developing pneumonia in this population and also specifically for developing viral pneumonia [23].

Within the entire cohort there was no association between the season in which an episode of pneumonia occurred and the severity of the pneumonia. However, RSV infection was highly seasonal with the peak occurring just after the wet season. Interestingly all of the infants who had an episode of RSV-associated pneumonia at less than two months of age were born in the wet season and were significantly more likely to have severe disease, making this group an important target for intervention [15]. However, the incidence of RSV associated pneumonia was lower in this age group possibly reflecting maternally acquired immunity.

Approximately 20% of children had multiple episodes of RSV associated pneumonia. This figure is similar to the number of multiple RSV associated infections reported by Nokes et al. However, in this report multiple episodes of RSV occurred in the same patient in the same RSV season [12], whereas in our study all repeat episodes occurred in subsequent RSV seasons and were not associated with more severe disease. This could reflect the increasing age of the child or could suggest the presence of increased immunity from the previous episode, possibly due to the high burden of RSV associated pneumonia in our population.

Secondary bacterial infection has been described in children with RSV associated pneumonia. Mahdi et al showed that, in HIV-unaffected children, a nine-valent pneumococcal conjugate vaccine prevented 32% (95% CI 6−50, p = 0.02) of RSV associated pneumonias [24]. This suggests that approximately one third of the RSV associated pneumonia episodes had a secondary infection caused by *Streptococcus pneumoniae*. In our study, we found a similar proportion of cases with suspected secondary bacterial infection: 30.1% of children had primary endpoint pneumonia on their CXR, which has been suggested to reflect a bacterial infection [25].

In a logistic regression model, children and infants with RSV associated pneumonia were more likely to have a fever on admission, tachycardia (as defined by appropriate age cut offs) and bilateral chest signs. These clinical features are not currently included in the WHO definition of pneumonia. While the success of the IMCI has been demonstrated, it is also evident that it has the potential to lead to the overuse of antibiotics [26]. For example, a recent study from Bangladesh showed that, in children 2–59 months of age, placebo was as effective as amoxicillin for treatment of non-severe pneumonia [27]. This could reflect that these cases had a viral rather than bacterial aetiology or were a mild bacterial infection that did not require treatment. Given the global increase in antibiotic resistance, thought should be given on how to use antibiotics more rationally. Unfortunately, in our study, although there was a significant association between RSV associated pneumonia and bilateral chest signs, the positive predictive value was only 37.4% (95% CI 33.8–41.2) and the negative predictive value 74.5% (96% CI 69.5–79.0). Therefore, on its own, the presence of bilateral chest signs would not be useful in determining whether a child should receive antibiotics during a respiratory illness. The presence of possible mixed bacterial viral infections also makes the decision to treat with antibiotics more difficult [28]. In our study we found that potentially one third of the RSV associated pneumonias seen could have had a secondary bacterial infection.

A limitation of our study was that we only looked at the severe end of the RSV disease spectrum, i.e. children with clinically defined pneumonia. This meant that we were unable to estimate the total incidence of RSV infection in our study population. However we were able to determine that RSV was a common cause of WHO defined pneumonia and, although it caused severe disease, this was most likely to be in younger children. We also demonstrated that there are clinical signs that make the diagnosis of RSV associated pneumonia more likely.

### Conclusion

RSV associated pneumonia is responsible for a significant proportion of clinical pneumonia episodes in young refugee children on the Thailand-Burma border. Interventions to prevent RSV infection have the potential to reduce the number of clinical pneumonia episodes diagnosed using the WHO criteria and hence reduce unnecessary antibiotic use.
